# The Relationship Between Psychotic Experiences and Sexual Risky Behaviors: Moderating Effects of Childhood Trauma and Depression in Population-Based Young Adults from Tunisia

**DOI:** 10.3390/healthcare14030332

**Published:** 2026-01-28

**Authors:** Feten Fekih-Romdhane, Emna Maalej, Majda Cheour, Frederic Harb, Souheil Hallit

**Affiliations:** 1 The Tunisian Center of Early Intervention in Psychosis, Department of Psychiatry “Ibn Omrane”, Razi Hospital, Manouba 2010, Tunisia; 2 Faculty of Medicine of Tunis, Tunis El Manar University, Tunis 1068, Tunisia; 3 Faculty of Medicine and Medical Sciences, University of Balamand, Kalhat, Tripoli P.O. Box 100, Lebanon; 4 School of Medicine and Medical Sciences, Holy Spirit University of Kaslik, Jounieh P.O. Box 446, Lebanon; souheilhallit@usek.edu.lb; 5 Applied Science Research Center, Applied Science Private University, Amman P.O. Box 11931, Jordan

**Keywords:** psychotic experiences, sexual risky behaviors, childhood trauma, depression, moderation

## Abstract

**Background/Objectives**: There is still limited understanding of how psychotic symptoms and sexual risky behaviors (SRBs) are related to each other. Gaining more knowledge of the mechanisms involved in this relationship could inform interventions to reduce or prevent SRBs. This study aims to deepen comprehension of the relationship between psychotic experiences (PEs) and SRBs by examining the moderating effects of depression and childhood trauma. **Methods**: A web-based survey and a cross-sectional design were adopted to collect data from 466 young general population adults (aged 18–35 years) from Tunisia during the period January–March 2024. The snowball sampling technique was used to recruit participants. **Results**: Moderation analyses were adjusted over age, sex, household crowding index, marital status, and living situation. The interaction PEs by childhood trauma was significantly associated with SRB scores. At high and moderate levels of child abuse, higher PEs were significantly linked to higher SRBs. Furthermore, the interaction PEs by depression was significantly associated with SRB scores. At high, moderate, and low levels of depression, higher PEs were significantly associated with higher SRBs. **Conclusions**: Clinicians should consider including assessment of childhood trauma and depression in young adults with PEs who are engaged in sexual risk-taking behaviors. Findings may imply that strategies addressing these two factors can be effective in mitigating the association between PEs and SRBs.

## 1. Introduction

Sexual risky behaviors (SRBs) entail a multitude of practices, including having multiple and/or high-risk sexual partners; engaging in unprotected vaginal, anal, or oral sexual intercourse; and early sexual debut [1,2]. In other terms, SRBs refer to objective, risky sexual behaviors, and not to emotional behaviors (such as sex used for emotion regulation). SRBs are widespread in the general population, with increasing trends of occurrences in low- and middle-income countries (LMICs) [3]. For instance, studies among youth from Arab countries, such as Palestine [4], Lebanon [5], and Tunisia [6], observed elevated proportions of unprotected sex, irregular or lack of condom use, and sex with multiple partners. Such unsafe sexual activities expose the individual to multiple harmful consequences, including infections with blood-borne viruses [7,8], unplanned pregnancy [9], forced sex [10], intimate partner violence [11], and more transactional sex [12]. SRBs may also lead to poor mental health outcomes [13,14,15]. Hence, SRBs are considered as an increasing public health problem [16], an important cause of burden of disease [17], and their prevention needs to be recognized as a public health priority [16].

Deepening understanding of the factors that may increase or decrease SRBs could help in guiding more effective health promotion interventions. Earlier research has shown that the reasons behind the decision to engage in SRBs in the youth population are multi-determined and result from complex interactions of many factors on multiple levels [18]. One possible factor reported by the literature as being associated with increased vulnerability to SRBs, which was chosen to be the focus of the present study, is psychotic experiences [19].

### 1.1. The Relationship Between Psychotic Experiences and SRBs

Since the 1990s, clinical studies have shown that individuals diagnosed with psychiatric illnesses are more susceptible to engage in SRBs, with those having a psychotic disorder being classified in the top ranks [20], and that people with advanced stages of psychotic disorders are more at risk of engaging in SRBs than the general population [21]. In particular, patients with schizophrenia who had psychotic experiences had a three-fold increased risk of having SRBs relative to those without psychotic experiences [19]. This has led authors to conclude that to address SRBs “emphasis should be given for patients who present with severe positive symptom” [19]. Greater psychotic experiences were also found to be associated with a nearly three times higher likelihood of having multiple sex partners [22]. More recently, similar evidence has been demonstrated in individuals with earlier stages of the disease. Young people with a first episode of psychosis have a 3.9 times higher likelihood of reporting unprotected sexual intercourse (i.e., inconsistent condom use) compared to their peer controls [23]. In this specific group, Brown et al. [24] found some evidence that psychotic symptoms may impact SRBs and suggested that the earliest clinical stages of psychotic disorders represent a pivotal time that offers a unique window of opportunity to shape better sexual health. At the same time, people with psychosis are more prone to experiencing health inequalities and disparities [25], including inequalities and disparities in sexual health [26]. Recently, a qualitative study identified a range of barriers to access to reproductive and sexual health care for people with emerging psychosis, including psychotic experiences per se and a lack of considering SRBs within early psychosis intervention care [27].

Despite all this evidence, two major gaps can be identified in the literature. First, there remains limited understanding of how psychotic experiences and SRBs are related to each other. Gaining more knowledge of the mechanisms involved in this relationship could inform interventions to reduce or prevent SRBs. Second, the studies available mostly included patients in the severe stages of the disease, while only scant research focused on individuals at the lowest end of the psychosis continuum (i.e., with psychotic experiences), where psychotic symptoms do not cross the threshold of severity for clinical psychotic disorder diagnosis. Psychotic experiences are relatively commonly represented in the general population, particularly during adolescence and young adulthood [28] and are distressing, debilitating, and enhance the risk of subsequent psychotic and other mental diseases [28,29]. We could find only one earlier study that empirically addressed SRBs in young people with psychotic experiences. Data from the 1990 British National Survey of Sexual Attitudes and Lifestyles revealed that childhood psychotic experiences (at age 12) were linked to a heightened risk of SRBs in young adulthood (at age 18) [30]. It is of note that SRBs can be identified by the presence of at least two of the following self-developed items: having sex after drugs/alcohol, sexual intercourse before age 16, having had multiple sexual partners with inconsistent use of contraception, having had a pregnancy, and having contracted a sexually transmitted disease [30]. Enhancing knowledge on how people with psychotic experiences may be at greater risk of SRBs may inform earlier and more effective risk-reduction measures and prevention strategies after the first emergence of psychotic experiences.

To bridge the two gaps mentioned above, this research sought to expand understanding of the nature and mechanisms of the relationship between psychotic experiences and SRBs. Although psychotic experiences are expected to have a significant effect on SRBs, this relationship may not be uniform across individuals. In this study, childhood trauma and depression are examined as moderators because theory and prior evidence suggest that they may shape how individuals respond to psychotic experiences, thereby affecting the strength of their association with SRBs.

### 1.2. Childhood Abuse and Depression as Potential Moderators

**Figure 1:**
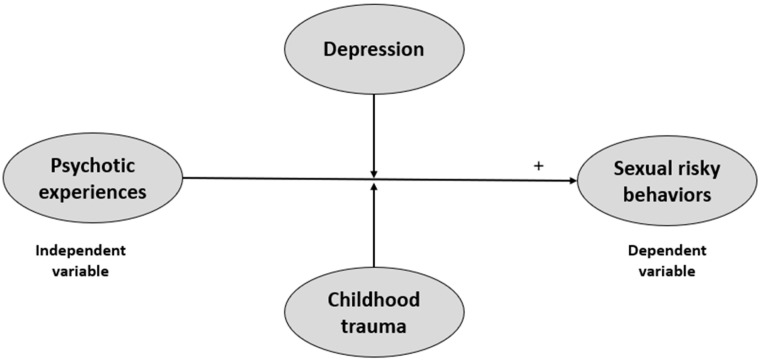
The conceptual moderation framework for the relationship between psychotic experiences and sexual risky behaviors, with depression and childhood trauma as moderators.

Our study intended to add to the existing literature by exploring a theoretically driven research framework (Figure 1) which posits that childhood trauma and depression may be potential moderators of the link between psychotic experiences and SRBs. As for depression, there is strong evidence from research among youth from across the world that depressive symptoms are associated with SRBs, such as multiple sexual partners, inconsistent condom use, and HIV infection [31,32,33,34,35]. For example, a recent study in a large sample of Swedish young adults (aged 18–30 years) found that high depression was significantly and positively related to 1.3 and 1.4 higher odds of not using a condom with a casual partner and having multiple sexual partners, respectively [14]. Another study among Finnish mid-teens showed a significant link between depression and non-use of condoms (and other contraceptives) and multiple sex partners [36]. In Ethiopia, moderately depressed students were almost two times more likely to engage in SRBs compared to their non-depressed counterparts [37]. Multiple explanations have been suggested to account for the occurrence of SRBs in depressed individuals, such as blurred judgement associated with depression that may impede the evaluation of the probable risks, or feelings of hopelessness and worthlessness that could drive these behaviors [38,39,40]. With regard to childhood trauma, an extensive amount of research has been dedicated to its association with multiple SRBs, such as engagement in transactional sex [41], early initiation of sexual behavior [41], and having sex with multiple partners [41,42]. In particular, sexual abuse was consistently shown to robustly predict several SRBs [43,44,45]. Other forms of childhood trauma, such as emotional/physical abuse [43,46,47] or neglect [47,48], were also demonstrated to have unique longitudinal effects on SRBs, even after adjusting for sexual abuse. In sum, earlier studies suggested that endorsement of a history of childhood adversity (of any form) seems to drive the risk for SRBs [49,50].

### 1.3. The Present Study

Adolescence (i.e., 10–24 years of age [51]) and young adulthood are particularly paramount for prevention, as these age ranges represent a period of peak onset for most mental health problems, including psychotic disorders [52]. In addition, youth of this age group exhibit the highest frequency of SRBs, as well as the greatest prevalence rates of unwanted pregnancies and STIs [14]. The present study proposes to build on the previous literature suggesting that numerous factors interact at different levels to decrease or increase SRBs, which might subsequently be targeted to guide effective health promotion interventions [18,53]. In particular, our study aimed to deepen comprehension of the relationship between psychotic experiences and SRBs by examining the moderating effects of depression and childhood trauma within a general population-based sample in Tunisia. Using cross-sectional data, it is hypothesized that more severe psychotic experiences will be associated with greater SRBs and that this association will be moderated by both depression and childhood trauma.

## 2. Materials and Methods

### 2.1. Sample and Procedure

A web-based survey and a cross-sectional design were adopted to collect data from young general population adults from Tunisia during the period January–March 2024. The snowball sampling technique was used to recruit participants. The eligibility criteria were as follows: (1) being a young community adult aged 18–35 years; (2) being of Tunisian origin and residency; (3) having no self-reported history of previous antipsychotic intake and/or psychotic disorders; and (4) being willing to participate and being able to understand the consent form. Specifically, the introductory paragraph of the survey introducing the study objectives was followed by a statement asking participants to confirm (yes or no), in order to be eligible, that they have no psychotic disorders previously diagnosed by a physician or a psychiatrist and/or no history of previous antipsychotic use. Participants were included regardless of whether they were currently sexually active or had a history of specific sexual acts. The survey took around 10 to 15 min to complete and was only available in the Arabic language. Prior to responding to the questionnaire, participants had access to the necessary information about the study. It was then made clear that informed consent to participate was provided when proceeding with the study. The protocol was approved by the ethics committee of Razi Hospital, Manouba, Tunisia. The study was conducted following the Declaration of Helsinki for human research. Confidentiality and anonymity were preserved.

### 2.2. Measurements

#### 2.2.1. Sociodemographic Data

Information on sex, age, marital status, and household crowding index (HCI; i.e., number of persons/number of rooms) was gathered from participants.

#### 2.2.2. The Sexual Risk Behaviors Scale (SRBS)

The SRBS is a recently developed and validated scale that assesses the subjective frequency of engagement in key sexual risk behaviors in the past month. It contains five items (e.g., “How often have you had sex without a condom with someone you have just met?”) [54]. Items are rated on a 5-point scale from 0 (“Never”) to 4 (“Very often”). The Arabic version of the scale was administered to participants [55] (Cronbach’s alpha = 0.86).

#### 2.2.3. The Prodromal Questionnaire—Brief (PQ-B)

The PQ-B is a self-report scale composed of 21 no/yes items [56,57] that was used in its Arabic validated version [58]. The PQ-B was developed as a screening tool, not a diagnostic tool. It evaluates psychotic experiences that are endorsed by help-seeking or non-help-seeking people within the general population, thus indicating risk for psychosis. The PQ-B has demonstrated good psychometric properties, with a Cronbach’s alpha = 0.95 in the present sample.

#### 2.2.4. Child Abuse Self Report Scale (CASRS-12)

The CASRS-12 is a shorter form of the Child Abuse Self Report Scale [59] that was validated in Arabic [60]. The scale is composed of 12 items divided into four dimensions: physical abuse (3 items, e.g., “During your childhood, did someone hurt you in a way that left bruises, marks, or injuries?”), psychological abuse (3 items, e.g., “As a child, did someone often belittle you, insult you, or make you feel worthless?”), sexual abuse (3 items, e.g., “Were you touched inappropriately by an adult when you were a child?”), and neglect (3 items, e.g., “During your childhood, did you frequently go without proper food, clothing, or shelter?”). Response options vary from 0 = never to 3 = always. Higher total scores reflect more severe childhood trauma (Cronbach’s alpha = 0.80).

#### 2.2.5. The Patient Health Questionnaire—9 (PHQ-9)

The PHQ-9 is a brief scale composed of nine items evaluating the severity of depressive symptoms [61] that was used in its Arabic validated version [62]. Each item is scored on a 4-point scale, with response options varying from 0 (“not at all”) to 3 (“nearly every day”). Higher scores designate more severe depression. In this study, the Cronbach’s alpha was 0.86.

### 2.3. Statistical Analysis

For the statistical analysis, the SPSS software v.25 was used. The skewness and kurtosis values outside the −2 and +2 window attested to the non-normal distribution of the SRB scores [63]. Consequently, we applied the log transformation to the score, which showed normal distribution. This transformation reduces positive skewness by compressing higher values more than lower values, thereby stabilizing variance and improving distributional symmetry. ANOVA for three means and Pearson’s test were used to correlate two continuous variables, and the Student’s *t*-test was used to compare two means. PROCESS MACRO (an SPSS add-on) v3.4 model 1 was used to conduct the moderation analysis [64], taking the childhood abuse and depression scores as moderators in the association between psychotic experiences and sexual risky behaviors. *p* < 0.05 was deemed statistically significant. The results of the moderation analysis were adjusted over variables that showed, in the bivariate analysis, a *p* < 0.25 [65].

## 3. Results

### 3.1. Sample Characteristics

Four hundred and sixty-six young adults (71.0% females) participated in this study. Our participants had a mean age of 24.60 ± 4.07 years. Other characteristics are shown in Table 1.

#### Bivariate Analysis of Factors Associated with Sexual Risky Behaviors

The findings indicated that male compared to female respondents, married compared to unmarried ones, and those who live alone compared to those living with family/partner or friend had significantly higher SRB scores (Table 2). Older age and higher child abuse and psychotic experiences were significantly linked to higher SRBs, whereas a greater HCI was significantly linked to lower SRBs (Table 3).

### 3.2. Moderation Analysis

**Figure 2:**
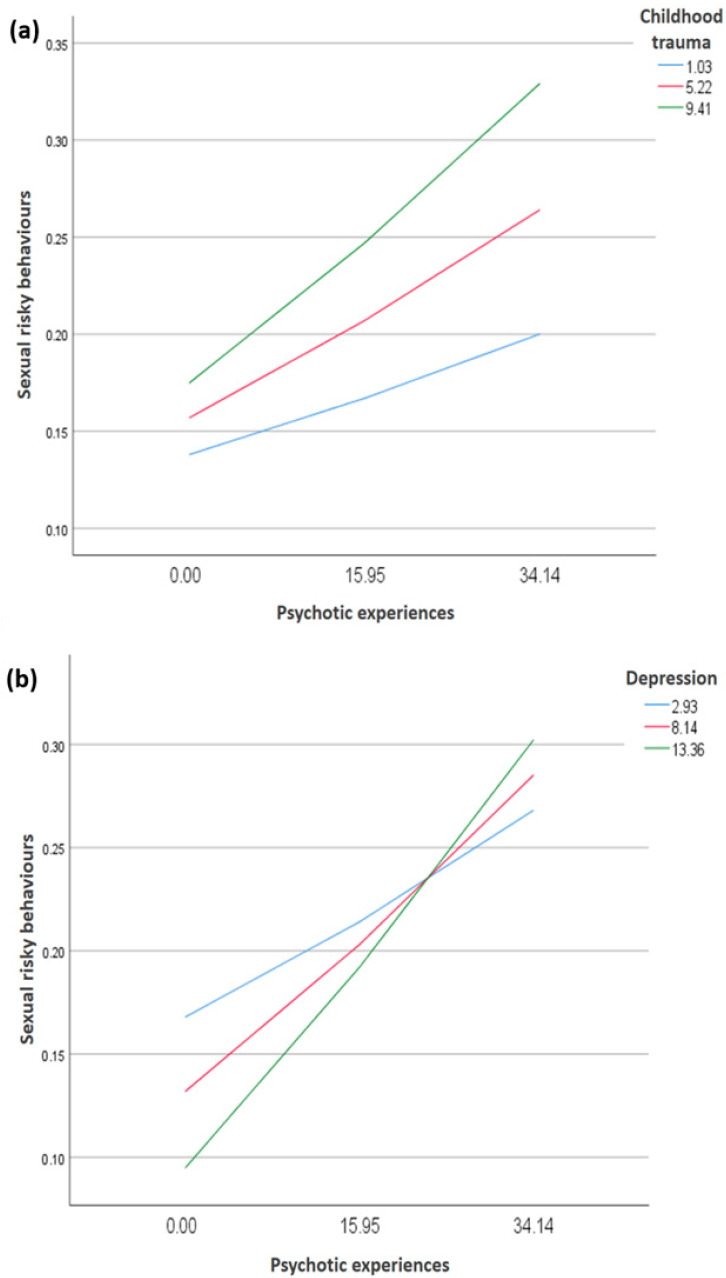
The moderation effect of childhood trauma (**a**) and depression (**b**) on the relationship between psychotic experiences and sexual risky behaviors.

Moderation analyses were adjusted over age, sex, HCI, living situation, and marital status. The interaction psychotic experiences by child abuse was significantly associated with SRB scores (Beta = 0.001; t = 2.89; *p* = 0.004; 95% CI < 0.001; 0.001). At high (Beta = 0.005; *p* < 0.001) and moderate (Beta = 0.003; *p* = 0.001) levels of child abuse, higher psychotic experiences were significantly associated with higher SRBs (Table 4, Model 1; Figure 2, right panel).

The interaction psychotic experiences by depression was significantly associated with SRB scores (Beta = 0.001; t = 2.35; *p* = 0.019; 95% CI < 0.001; 0.001). At high (Beta = 0.006; *p* < 0.001), moderate (Beta = 0.004; *p* < 0.001), and low (Beta = 0.003; *p* = 0.029) levels of depression, higher psychotic experiences were significantly linked to greater SRBs (Table 4, Model 2; Figure 2, left panel).

#### Stratification by Sex

In males, the interaction psychotic experiences by child abuse was significantly associated with SRB scores (Beta = 0.001; t = 2.06; *p* = 0.042; 95% CI < 0.001; 0.002). At high (Beta = 0.008; *p* < 0.001) and moderate (Beta = 0.004; *p* = 0.026) levels of child abuse, higher psychotic experiences were significantly associated with higher SRBs. The interaction psychotic experiences by depression was also significantly associated with SRB scores (Beta = 0.001; t = 2.94; *p* = 0.004; 95% CI < 0.001; 0.001). At high (Beta = 0.007; *p* = 0.003) levels of depression, higher psychotic experiences were significantly linked to greater SRBs.

In females, the interaction psychotic experiences by child abuse was significantly associated with SRB scores (Beta = <0.001; t = 2.44; *p* = 0.015; 95% CI < 0.001; <0.001). At high (Beta = 0.004; *p* < 0.001) and moderate (Beta = 0.003; *p* = 0.011) levels of child abuse, higher psychotic experiences were significantly associated with higher SRBs (Table 5, Model 3). The interaction psychotic experiences by depression was not significantly associated with SRB scores (Beta = <0.001; t = 0.99; *p* = 0.325; 95% CI < 0.001; <0.001).

## 4. Discussion

This study is, to our knowledge, amongst the first to explore the relationship between psychotic experiences and SRBs and to examine if depression and childhood trauma moderate this association among general population young adults aged 18–35 years from an Arab country in the Middle East and North Africa (MENA) region. The study hypothesis was supported, and both factors were found to serve as significant moderators in the association between psychotic experiences and SRBs. These findings are an important and highly relevant contribution to the field, as research on SRBs is rather scant in this cultural context [66], hampered by multiple religious, cultural, and legal prohibitions. Indeed, SRBs are still taboo health subjects for individuals, clinicians, and researchers. Due to globalization and economic changes, there have been major societal changes in most of the Arab countries, such as a rise in the age of marriage [67]. However, cultural and religious norms (such as the forbidding of sexual activities outside of marriage) have not changed at the same pace and remain anchored in the population’s beliefs, attitudes, and practices [67]. It is therefore valuable to provide an overview of the factors related to SRBs in such cultural environments.

Our findings demonstrated that higher psychotic experiences are significantly associated with higher levels of risk-taking sexual behaviors. The conceptual framework of the present study was developed based on a sound theoretical background and an empirical basis which support that psychotic symptoms are likely associated with more engagement in SRBs in both clinical [19,22,23,24] and non-clinical [30] populations. Such observations have prompted many researchers to call for an urgent integration and improvement of the sexual and reproductive health care within early psychosis intervention programs [27]. At present, however, the majority of research on psychosis and SRBs has concentrated on populations in more advanced phases of the disease. Additionally, there is still a relatively poor understanding of the mechanisms underscoring the relationship between psychotic symptoms and engagement in risky sex.

Exploring moderators of this relationship at the mildest end of the psychosis continuum in a population-based sample can better inform effective prevention strategies and appropriate interventions. To this end, this study sought to test the moderating effect of two individual psychological factors, namely, depression and childhood trauma. Analyses showed that depression and childhood trauma served as significant moderators in the relationship between psychotic experiences and sexual risk-taking. In particular, at any level of depression and at moderate-to-high levels of childhood trauma, more severe psychotic experiences were associated with greater SRBs. These results concur with previous empirical findings suggesting that childhood trauma and depression play a crucial role in SRBs [47,68] and that both factors interplay significantly with psychotic symptoms within psychotic disorder populations [69,70]. From a theoretical perspective, the Psychosocial Stress Model posits that the stress (here, childhood trauma) associated with mental health problems (here, psychotic experiences) may foster the emergence of maladaptive coping mechanisms, including engaging in SRBs [71]. The positive association between psychotic experiences and SRBs may also depend on the presence of depression. Depression represents a key psychological factor that can amplify the experience of psychosis and provoke SRBs. According to the cognitive theory of depression, cognitive distortions lead to the appearance of symptoms of depression. Depressive symptoms may impair cognitive and social functioning, thereby amplifying the tendency of individuals with psychotic experiences to engage in high-risk behaviors [72]. The moderation analyses indicated that psychotic experiences were significantly associated with SRBs even at low levels of depressive symptoms. This finding suggests that depression does not initiate the relationship between psychotic experiences and SRBs but rather amplifies an association that is already present. In other words, depressive symptoms may sensitize individuals to the behavioral impact of psychotic experiences, resulting in stronger associations with SRBs as depressive severity increases. Altogether, our results suggest that targeted interventions that specifically focus on these moderators may have downstream effects on involvement in SRBs. The small numerical size of the regression coefficients observed in the moderation analyses (Beta = 0.001–0.006) is partly attributable to the logarithmic transformation applied to the SRB score. Log-transformed dependent variables yield coefficients that reflect proportional changes rather than absolute increases in the original scale, resulting in attenuated Beta values. Accordingly, the practical significance of these findings lies not in the absolute size of the coefficients, but in the consistent pattern showing that higher levels of child abuse and depressive symptoms amplify the association between psychotic experiences and SRBs.

### 4.1. Limitations

There are limitations to this study that should be acknowledged. First, only subjective self-administered measurements were adopted instead of more objective measures, which can be subject to social desirability and recall biases. Second, it only included individuals from Tunisia, which could hinder the generalizability of our results to the larger Arab community. Earlier evidence suggested that sexual risk behaviors may be culturally influenced [73]. Tunisia has a conservative culture and society, where sexual norms are largely shaped by taboos and religious restrictions [74]. This highlights that the Tunisian-specific context might have influenced the results and that our conclusions may not easily apply to other culturally distinct cultures. Third, the cross-sectional design precluded conclusions on causality. Future longitudinal research is warranted to confirm the present findings and establish causal connections between variables. Fourth, the reliance on an online survey design and snowball sampling through social networks may have reduced sample heterogeneity and introduced a risk of selection bias, especially as the sample was predominantly female (71.0%). This approach may also have resulted in the overrepresentation of individuals who are more engaged with the study topic, more digitally literate, or more connected to social media. Therefore, our results should be interpreted with caution and may not be fully generalizable to the target population. To enhance representativeness, future research should consider probability-based recruitment strategies. Fifth, the SRBS assesses SRBs only in the past month, which limits the generalization of the results to long-term patterns. Residual bias is possible since some factors were not taken into consideration in the analysis (substance use, socioeconomic status beyond HCI, religiosity, and sexual orientation), which might affect interpretability. Finally, other potential mediators/moderators were not tested, such as behavioral factors (e.g., alcohol use), family factors (e.g., socioeconomic status), and socio-environmental factors (e.g., peer, school, and community influences) [75]. These factors still need to be considered in further studies.

### 4.2. Clinical and Research Implications

SRBs place young adults at risk for detrimental physical/mental health and social outcomes. This risk seems to be further heightened in vulnerable youth who experience psychotic experiences. In other words, the findings indicate that psychotic experiences are closely and positively related to SRBs, which implies that prevention and intervention efforts could target young adults who have psychotic experiences as a high-risk group for elevated SRB problems. Nevertheless, some evidence indicates that there are multiple barriers to mental health clinicians discussing reproductive and sexual health with people with early psychosis, such as discomfort, perception of sexual health as taboo, lack of training, prioritization of mental health, and programmatic barriers [27]. Clinicians’ denial of early psychosis patients’ sexuality may negatively influence the treatment and hinder the achievement of overall wellbeing [76]. We hope that our findings will draw attention to these issues and encourage clinicians who work with young people in early intervention in psychosis services to routinely evaluate and address SRBs. In light of our findings, it is also recommended to pay particular attention to the possible existence of psychotic experiences in young people who consult for sexual ill health. Furthermore, clinicians should consider including assessment of childhood trauma and depression in young people with psychotic experiences who engage in sexual risk-taking behaviors. Implementation of evidence-based intervention strategies that address these two factors (e.g., psychotherapy and Trauma-Focused Cognitive Behavioral Therapy [77]) can be effective in mitigating the relationship between psychotic experiences and SRBs.

## 5. Conclusions

This research showed that individuals with moderate–high childhood trauma histories and/or low–moderate–high depressive symptoms may be more likely to engage in SRBs, particularly when they have psychotic experiences. Our results in an underrepresented population and culture hold the promise of enriching the psychosis and sexual health fields. However, given the cross-sectional design and the self-reported nature of the data, caution should be used when interpreting the present findings. Future longitudinal and experimental research that sheds a brighter light on the causal relationship between psychotic experiences and SRBs and their underlying mechanisms is highly encouraged, as it could be highly relevant from a public health perspective for intervention and early prevention.

## Figures and Tables

**Table 1:** Sample characteristics (N = 466).

Variables	N (%)
Sex	
Female	331 (71.0%)
Male	135 (29.0%)
Marital status	
Married	53 (11.4%)
Unmarried	413 (88.6%)
Living situation	
With friends	81 (17.4%)
With family/partner	333 (71.5%)
Alone	52 (11.2%)
**Variables**	**Mean ± SD**
Age (years)	24.60 ± 4.07
HCI	1.07 ± 0.45
Sexual risky behaviors raw score	1.89 ± 3.34
Sexual risky behaviors log score	0.22 ± 0.35
Childhood trauma	5.22 ± 4.19
Depression	8.14 ± 5.21
Psychotic experiences	15.95 ± 18.19

HCI: household crowding index.

**Table 2:** Bivariate analysis of variables linked to sexual risky behaviors.

Variable	Mean ± SD	*t*/*F*	*df*/*df1*,*df2*	*p*
Sex		3.81	464	**<0.001**
Male	0.32 ± 0.38			
Female	0.17 ± 0.32			
Marital status		−2.47	464	**0.014**
Single	0.20 ± 0.35			
Married	0.33 ± 0.34			
Living situation		5.15	2, 463	**0.006**
With family/partner	0.19 ± 0.32			
With friends	0.22 ± 0.38			
Alone	0.36 ± 0.41			

Numbers in bold indicate significant *p* values. The log SRB score was used for the analyses.

**Table 3:** Pearson correlation matrix.

	1	2	3	4	5
1. Sexual risky behaviors	1				
2. Age	0.31 ***	1			
3. HCI	−0.15 **	−0.20 ***	1		
4. Child abuse	0.25 ***	0.09	0.08	1	
5. Depression	0.09	−0.06	0.01	0.36 ***	1
6. Psychotic experiences	0.21 ***	−0.16 **	0.09	0.37 ***	0.46 ***

** *p* < 0.01; *** *p* < 0.001; HCI: household crowding index.

**Table 4:** Conditional effects of the focal predictor (psychotic experiences) at values of the moderators in the total sample.

	Beta	t	*p*	95% CI
**Model 1: Child abuse as the moderator**				
Low (=1.03)	0.002	1.60	0.111	<0.001, 0.004
Moderate (=5.22)	0.003	3.50	**0.001**	0.001, 0.005
High (=9.41)	0.005	5.11	**<0.001**	0.003, 0.006
**Model 2: Depression as the moderator**				
Low (=2.93)	0.003	2.19	**0.029**	<0.001, 0.006
Moderate (=8.14)	0.004	4.66	**<0.001**	0.003, 0.006
High (=13.36)	0.006	6.22	**<0.001**	0.004, 0.008

Numbers in bold indicate significant *p* values.

**Table 5:** Conditional effects of the focal predictor (psychotic experiences) at values of the moderators stratified by gender.

	Beta	t	*p*	95% CI
**Male sex**				
**Model 1: Child abuse as the moderator**				
Low (=1.28)	0.001	0.39	0.695	−0.004; 0.006
Moderate (=5.24)	0.004	2.25	**0.026**	0.001; 0.008
High (=9.21)	0.008	3.38	**0.001**	0.003; 0.012
**Model 2: Depression as the moderator**				
Low (=2.41)	−0.002	−0.63	0.531	−0.008; 0.004
Moderate (=7.36)	0.002	1.02	0.309	−0.002; 0.007
High (=12.31)	0.007	3.07	**0.003**	0.002; 0.011
**Female sex**				
**Model 3: Child abuse as the moderator**				
Low (=0.92)	0.001	1.15	0.252	−0.001; 0.004
Moderate (=5.21)	0.003	2.57	**0.011**	0.001; 0.004
High (=9.50)	0.004	3.89	**<0.001**	0.002; 0.006

Numbers in bold indicate significant *p* values.

## Data Availability

The datasets generated and/or analyzed during the current study are not publicly available due to restrictions from the ethics committee but are available from the corresponding author [SH] on reasonable request.

## Abbreviations

The following abbreviations are used in this manuscript:

SRBs	Sexual risky behaviors
HCI	Household crowding index
